# Total productivity change of Health Centers in Greece in 2016–2018: a Malmquist index data envelopment analysis application for the primary health system of Greece

**DOI:** 10.1186/s12962-021-00326-z

**Published:** 2021-11-02

**Authors:** Anastasios Trakakis, Miltiadis Nektarios, Styliani Tziaferi, Panagiotis Prezerakos

**Affiliations:** 1 Faculty of Health Sciences, University of Peloponnese, Tripoli, Greece; 2 Faculty of Finance and Statistics, University of Piraeus, Piraeus, Greece

**Keywords:** Health Centre’s Total Productivity, Data envelopment analysis, Malmquist productivity index, Internal and External Validity, Primary Health Care, C14, C32, C52, I10

## Abstract

**Background:**

This paper attempts to evaluate the primary health care system by evaluating health centres in Greece.

**Methods:**

Malmquist Index Data Envelopment Analysis is applied to study the total productivity of 155 health centres in Greece during 2016–2018. The data were collected from the Ministry of Health and submitted to quality tests to ensure validity and avoid bias.

**Results:**

This paper measures the productivity of each of the 155 health centres in Greece and how it shifted during 2016–2018. In addition, the overall productivity change of the 155 health centres over time is calculated and analysed as being due to technical efficiency or technological efficiency. The analysis of the mean values showed a decrease of 0.9% in the overall productivity factor from 2016 to 2017 and a decrease of 5.2% from 2017 to 2018. The overall decrease in the productivity of the 155 health centres was 3.1%. From 2016 to 2018, 59 health centres changed their productivity mainly due to technological change and 91 changed mainly due to technical efficiency change. One health centre showed regression to its total productivity due to equal regression of its technical efficiency and technology.

**Conclusions:**

The method used is nonparametric data envelopment analysis along with the Malmquist index to include panel data in the analysis. Meaningful results were extracted by indicating the number of health centres that improved their productivity, regressed in productivity, or remained constant through the period 2016–2018. This paper may contribute to improving health centres’ efficiency and productivity. Furthermore, valuable results can be extracted for the National Health Care System to match available resources that correspond to each health centre’s needs, as well as for manager planners and stakeholders in primary health care.

**Supplementary Information:**

The online version contains supplementary material available at 10.1186/s12962-021-00326-z.

## Background

Primary health care has recently received more attention, as the World Health Organization (WHO) has called attention to its importance to the population’s overall health and its contribution to the National Health System of every country. Every country in the OECD participates in a strategy that focuses on strengthening primary health care, as stated by WΗΟ at Alma-Ata in 1978. OECD countries, including Greece, attempted to monitor primary health care to follow the strategy imposed by the WHO [1].

In 2013, the health care expenditures in Greece were higher than the average expenditures of OECD Countries by 0.3%. The economic crisis led to funding cuts in health care services, reducing health care expenditures below the average of OECD countries by 1.8% in 2017, intensifying the inadequate primary health care system and delivery of health care services [2–4]. The lack of health promotion, disease prevention, and rehabilitation as well as the dissatisfaction of people receiving health services indicate distribution problems in health services, problematic administration, and low productivity. This is mainly due to the inadequacy of primary health care, which highlights the importance of its contribution to the whole health care system and the efforts that should be made to strengthen it [5–7]. Moreover, the necessity of a stronger and integrated primary health care system is indicated by the fact that Greece faces one of the highest rates of chronic diseases among the EU-27, as well as one of the highest overall household health expenditures [8].

The economic crisis of the last decade in Greece was accompanied by funding cuts for the National Health System. The bailout program by international creditors and the three Economic Adjustment Programs led to additional funding cuts for the public sector and Primary Health Care System between 2010 and 2018 [9]. To overcome the negative effects of funding cuts while strengthening the National Health System, reforms were introduced. Emphasis was placed on primary health care and its contribution to the population’s overall health. The national strategy of the reform involved establishing the National Primary Health Care Networks (PEDY) in 2014. All public Primary Health Care services were transferred to PEDY and coordinated by Regional Health Authorities (ESY), which was established to create a two-pillar system from 2017 to 2020. Law 4486/2017 introduced the reform of primary health care, including radical changes with the establishment of local health units (TOMY) and contracted family doctors, as the first point of providing health services and guiding people through the health system. The new primary health care model focuses on prevention, health promotion, and integrated care [8, 9].

The reform included establishing 239 TOMYs representing the first pillar of primary health care and the upgradation of health centres representing the second pillar of primary health care. Therefore, health centres would include more complex health services. Moreover, each TOMY would be under the supervision of a corresponding health centre. This reform is based on the principles of primary health care and aims to establish an integrated model of the primary health care system [9]. Eventually, 127 TOMYs opened, which were mostly concentrated in mainland Greece. Therefore, rural areas were barely covered, while the urban population bypassed TOMY with direct access to hospitals or the private sector [8].

This paper attempts to evaluate the efficiency of health centres in Greece during the years of the major reform, which coincided with the last years of economic adjusted programs.

Health centres are one of the main compounds of primary health systems. By attempting to evaluate their productivity and efficiency, important information would be brought to light regarding primary health care in Greece. In 2016–2018, 207 health centres were operating in Greece, of which 155 were submitted for this paper’s analysis. The other 52 health centres were excluded due to lack of data to avoid random estimation and possibility of bias. Out of the 155 health centres, 9 were operating in the provision of Attica, which is the main urban area of Greece. In addition, 5 health centres were operating in semiurban areas. All the other health centres submitted to the paper's analysis were operating in rural areas (90%).

## Method

There are two approaches to measuring efficiency according to the relevant literature, parametric and nonparametric. Nonparametric approaches are most commonly used in health care studies since they have the advantage that the functional form does not need to be known [10]. The most commonly used nonparametric approach is data envelopment analysis (DEA) [11]. Moreover, DEA has the advantage of handling multiple inputs and outputs and is used with any input–output measurement. In studies measuring health care efficiency, DEA is the most commonly used method [12].

Farrell (1957), based on Debreu (1951) and Koopmans (1951), first introduced modern efficiency and attempted to measure the efficiency of a firm by considering multiple inputs [13, 14]. Farrell analysed and divided the efficiency measured into two components, technical and allocative efficiency, both of which appraise economic efficiency [15].

Charnes, Cooper and Rhodes (CCR, 1978), based on Farrell, proposed the DEA model. DEA is a linear programming method that constructs a nonparametric frontier containing all the firms submitted for analysis to measure their efficiencies. The firms are called decision-making units (DMUs), and their data, translated as inputs and outputs, are used to measure their efficiencies. The constructed frontier includes all efficient DMUs, while below the frontier, all inefficient DMUs are placed. Technical efficiency depends on the “input–output ratio of productivity” [16] and can be divided into pure technical and scale efficiency. Essentially, technical efficiency refers to converting inputs into outputs according to best practice so that the DMU is as efficient as possible [2].

Technical efficiency measured by DEA has two orientations according to the relevant literature. The input orientation of DEA binds the outputs produced to solve a linear programming equation that minimizes the inputs used. In contrast, the output orientation of DEA binds the inputs used to solve a linear programming equation that maximizes the outputs produced [10, 17].

In addition, two methods of DEA have been proposed: the first is based on the assumption of constant return to scale (CRS), as was introduced by Charnes, Cooper and Rhodes (CCR, 1978), while the other is based on the assumption of a variable return to scale (VRS), as later introduced by Banker, Charnes and Cooper in 1984. “The CRS method is applied when all DMUs are operating at an optimal level while under imperfect competition, the VRS method is applied considering that not all DMUs are operating at an optimal level, assuming that there are scale efficiencies” [18, 19].

CRS and VRS DEA can each measure the technical efficiencies of the DMUs included in the analysis, but they are not able to utilize panel data, means they are not able to measure the impact of time on the efficiencies. To overcome this limitation, the Malmquist productivity index (MPI) can be used along with the DEA. The MPI was first introduced by Caves et al. (1982), who relied on Shephard’s (1970) distance function to measure the productivity change over time and divide it into change due to technology and technical efficiency [20].

Productivity is defined as the ratio of an index of outputs over an index of inputs used to produce them [21–23]. Increasing productivity means that more outputs are obtained from the same amount of inputs, or fewer inputs are required to produce the same output. The change in productivity over time is called productivity change and shifts over time due to technical efficiency and technical/technological change [2, 15, 24–26].

MPI DEA is essentially a nonparametric mathematical programming approach, which, according to the literature, is the most widely used method to include panel data in the analysis and calculate the indices of total factor productivity, technical efficiency, and its components (pure technical efficiency and scale efficiency) and technological change over time [2]

There are many applications of MPI DEA in health care studies. In Greece, Xenos et al. [27] measured the efficiency and productivity assessment of public hospitals in Greece during the crisis period 2009–2012. Additionally, Dimas et al. [11] measured productivity performance and its components in Greek public hospitals. In contrast, Androutsou et al. [2] measured efficiency and productivity across hospitals in the Regional Health Authority of Thessaly, Greece. Fragkiadakis et al. [28] measured the operational and economic efficiency of public hospitals in Greece. In contrast, this study, although it uses MI DEA, focuses on primary health care in Greece, which has recently gained more interest, and there is a lack of applications measuring its efficiency and productivity.

In this paper, the input-oriented MPI DEA is used. The first reason for this is because in the health sector, it is impossible to predefine outputs, but inputs can be predefined and controlled. Second, MPI DEA is used because panel data are included in the paper [29–31].

## Model specification—data envelopment analysis and Malmquist productivity index

The mathematical concepts of DEA and MPI are briefly analysed below since the aim of this paper is to evaluate the productivity and efficiency of the 155 health centres in Greece during 2016–2018. An extended mathematical analysis of the methods of DEA and MPI and how they are used is presented in the relevant literature.

Before presenting the mathematical background of MPI, DEA will be analysed. The mathematical analysis refers to an input-oriented DEA under the CRS and VRS assumptions, since both are needed to estimate MPI.

In the input-oriented mathematical model of CRS, it is assumed that there are “N DMUs that use K inputs to produce M outputs. Under this assumption, there are two matrices, the K*N input matrix, referred to as the X, and the M*N output matrix, referred to as Y, which both represent the data of all N DMUs” [32]. To measure the efficiency of the DMU, the literature considers the calculation of the ratio of all outputs over all inputs. T.J. Coelli presented the following mathematical linear programming problem in 1996:

“min_θ,λ_ θ,

s.t.

−y_i_ + Yλ ≥ 0,

θx_i_-Xλ ≥ 0,

λ ≥ 0.

The symbol θ is a scalar, and λ is an N*1 vector of constants” [32]. The symbol θ represents the efficiency of the DMUs, and their values are within a closed interval of (0,1). The problem aims to compute the values of θ. Values of 1 mean that the DMU operates at an optimal level of efficiency, while values less than 1 mean the DMUs performance is inefficient. The mathematical function has to be solved N times for each DMU [32].

The CRS model is based on the assumption that all DMUs operate at an optimal scale. In contrast, the VRS model avoids this assumption, because there might be scale efficiencies. By adding the constraint N1’λ = 1 to the CRS model, scale efficiency effects are calculated, and technical efficiency is divided into pure technical efficiency and scale efficiency for each DMU [19]. N1 represents an N*1 vector of ones [32].

The MPI is an extended application of DEA to measure productivity change over time for each DMU and analyse it into change owing to technical efficiency and change owing to technology [32].

MPI DEA is used for panel data, and there is no need to choose between the CRS or VRS approach since they give the same results. In estimating the MPI DEA, both the CRS and VRS approaches are used to calculate the various distances that construct the Malmquist indices [33].

There are two orientations for the MPI DEA method: input and output orientations. In the input orientation, the production is described by calculating the minimal proportional decrease of the input vector, given the output vector. In contrast, in the output orientation, the production is described by calculating the maximal proportion increase of the output vector, given the input vector [34].

In this paper, panel data for three years (2016–2017–2018) are considered, forming two periods (2016–2017 and 2017–2018). The calculation of MPI involves the estimation of four distances for each of the two periods. DEA analysis by CRS and VRS assumptions is performed for each of the three years (2016, 2017 and 2018), measuring the efficiencies of the DMUs. Note that although the efficiencies of the DMUs for 2016 are calculated, Malmquist indices cannot be estimated, as no data are available for 2015.

The Malmquist index was first introduced in 1970 with Shephard’s distance function and has since been widely used in many areas where efficiency needs to be measured with panel data. Färe specified an output-oriented MPI in 1994 [35]. In this paper, the input-oriented MPI is used.

The MPI for a period (t to t + 1) represents the productivity at the production point (x_t+1_, y_t+1_) relative to the production point (x_t_, y_t_) for each DMU.

“M (y_t+1_, x_t+1_, y_t_, x_t_) = {[d^t+1^ (x_t+1_, y_t+1_)/d^t^ (x_t_, y_t_)]* [(d^t^ (x_t_, y_t_)/d^t+1^ (x_t_, y_t_))*(d^t^ (x_t+1_, y_t+1_)/d^t+1^ (x_t+1_, y_t+1_))]^1/2^}” [24, 32, 35, 36].

The first fraction of the equation represents technical efficiency change, while the second fraction represents technology change for the period (t to t + 1). Moreover, technical efficiency change can be further analysed into change due to pure technical efficiency and scale inefficiency. Positive total productivity growth from time t to time t + 1 means a value greater than one for the index [24, 32, 35, 36].

To calculate the MPI equation, the four distances mentioned above must be calculated by linear programming methods.

1. [d^t^ (x_t_, y_t_)]^−1^ = min_θ,λ_ θ, s.t. − y_it_+Y_t_λ≥0, θx_it_–X_t_λ≥0, λ≥0
2. [d^t+1^ (x_t+1_, y_t+1_)]^−1^ = min_θ,λ_ θ, s.t. − y_i,t+1_+Y_t+1_λ≥0, θx_i,t+1_ − X_t+1_λ≥0, λ≥0
3. [d^t^ (x_t+1_, y_t+1_)]^−1^ = min_θ,λ_ θ, s.t. − y_i,t+1_+Y_t_λ≥0, θx_i,t+1_ − X_t_λ≥0, λ≥0
4. [d^t+1^ (x_t_, y_t_)]^−1^ = min_θ,λ_ θ, s.t. -y_i,t_+Y_t+1_λ≥0, θx_i,t_-X_t+1_λ≥0, λ≥0

Note that in this paper, N(3 T-2) linear programming equations must be calculated [30, 34]. Considering the 155 health centres and the 2 time periods that were included in the analysis, 620 linear programming equations need to be calculated.

## Data

Efforts were made to include all health centres of Greece in the analysis of this paper, but due to lack of data, only 155 health centres were included. Fifty-two health centres were excluded to avoid random estimation and the possibility of bias.

The sample used for the analysis of this paper is homogenous, as it includes the majority of health centres of Greece (74.87% of the total), distributed across the seven health regions of Greece. Out of the 155 health centres, 9 were operating in Attica, which is the main urban area of Greece. In addition, 5 health centres were operating in semiurban areas. All the other health centres submitted to the paper's analysis were operating in rural areas (90%). The 155 health centres use the same categories of inputs, generating the same categories of outputs, differing only in the quantities used. This ensures comparability and validates this paper in measuring the health centres’ productivity and efficiencies for the years 2016, 2017, 2018 with DEA. Furthermore, according to the literature, the requirements for conducting MPI DEA are satisfied, ensuring meaningful results. These requirements include that at least one DMU in the sample consumes and produces each input and output and that each DMU in the sample consumes at least one input and produces at least one output [37, 38]. By including the majority of health centres in Greece, discriminatory power between the efficient and inefficient units is also achieved [39, 40].

In the analysis of this paper, 12 outputs were included to measure the change in productivity and the change in technical efficiency and technology of each health centre. The outputs represent the total health care services provided by each health centre:

1. Total number of “Nursing Operations” applied—Output1
2. Total Number of “Microsurgeries” applied—Output2
3. Total Number of “Dental Procedures” applied—Output3
4. Total number of “Chronic disease cases” faced—Output4
5. Total number of “Emergencies” faced—Output5
6. Total Number of “Regular Incidents” faced—Output6
7. Total Number of “Urgent Incidents” faced—Output7
8. Total Number of “Transcriptions” given—Output8
9. Total Number of “Biopathological and Laboratory exams” applied—Output9
10. Total Number of “Test Mantoux” applied—Output10
11. Total Number of “Vaccinations for adults” applied—Output11
12. Total Number of “Vaccinations for kids and teenagers” applied—Output12

In contrast, 4 inputs were used, representing the total staff employed and occupied in the health centres:

(13) Total “Number of Managers” employed—Input1
(14) Total “Number of Doctors” employed—Input2
(15) Total “Number of Nursing Staff” employed—Input3
(16) Total “Number of non-medical staff” employed—Input4

All inputs and outputs used for this paper are for the years 2016, 2017 and 2018. In Additional File 1, a table is presented, which shows the descriptive statistics of all inputs and outputs used to evaluate the total productivity and efficiency of each of the 155 health centres included in the analysis of this paper. The descriptive statistics show the minimum, maximum and mean values, as well as the standard deviation of each input and output included in the analysis.

## Results

### Productivity

The productivity and efficiency of each of the 155 health centres were measured by performing input-oriented MPI DEA using the DEAP ver2.1 program.

The summary table with the results of technical efficiencies under the CRS and VRS assumptions for 2016, 2017, and 2018 of all DMUs included in the analysis is presented below (Table 1).

**Table 1:** Descriptive statistics, mean, maximum, minimum and standard deviation of the efficiencies in each year, under CRS and VRS assumption for the 155 DMU’s

	YEAR1-crs	YEAR1-vrs	YEAR2-crs	YEAR2-vrs	YEAR3-crs	YEAR3-vrs
Mean	0.85419	0.94366	0.85128	0.92827	0.85074	0.93104
Std. Deviation	0.203580	0.134536	0.210957	0.149666	0.197387	0.139425
Minimum	0.195	0.318	0.274	0.307	0.317	0.333
Maximum	1.000	1.000	1.000	1.000	1.000	1.000

Ν: 155, Valid: 155, Missing: 0

The Spearman’s rank correlation coefficient between the efficiencies calculated under the CRS and VRS assumptions is calculated for each year to interpret the correlation between the various rankings given by CRS and VRS to determine the degree of association between the 2 methods [41] (Table 2).

**Table 2:** Spearman-rank correlations between input-oriented crste and vrste models

Year 2016		
	YEAR1 CRS	YEAR1 VRS
YEAR1_CRS	1.000	0.571^**^
YEAR1 VRS	0.571^**^	1.000

Year 2017		
	YEAR2 CRS	YEAR2 VRS
YEAR2_CRS	1.000	0.698^**^
YEAR2 VRS	0.698^**^	1.000

Year 2018			
		YEAR3 CRS	YEAR3 VRS
YEAR3_CRS		1.000	0.665^**^
YEAR3 VRS		0.665^**^	1.000

**Correlation is significant at the 0.01 level (2-tailed). Ν:155

For 2016, 2017, and 2018, the correlation coefficients are 0.571, 0.698, and 0.665, respectively. The statistically significant coefficients show a high degree of correlation between the CRS and VRS methods for each year.

### Malmquist productivity index

The results for the 2016–2017 and 2017–2018 periods, as well as the overall results for the period 2016–2018, are presented in Additional File 2 for all the firms included in the analysis. Effch, techch, pech, sech and tfpch columns represent the indices related to efficiency change, technological change, pure technical efficiency change, scale efficiency change and total factor productivity change, respectively. Indices with values greater than one indicate progress for the health centres, values less than one indicate decline, and values equal to one indicate no change from t to t + 1 [34].

Table 3 shows summary statistics for all DMUs with their mean, minimum and maximum values, the Standard Deviation of effch, techch, and tfpch under each of the two periods, and the overall results for both periods.

**Table 3:** Arithmetic mean, minimum, maximum and St. deviation of effch, techch, tfpch of each period for all DMU’s

	effch_2016_2017	effch_2017_2018	effch_mean
Arithmetic mean	1.02483	1.04426	1.01459
Std. Deviation	0.283444	0.328508	0.182204
Minimum	0.336	0.323	0.600
Maximum	3.256	3.430	2.197

	techch_2016_2017	techch_2017_2018	techch_mean
Arithmetic mean	1.01713	0.96447	0.97400
Std. Deviation	0.220324	0.209529	0.104796
Minimum	0.580	0.270	0.609
Maximum	2.413	2.155	1.446

	tfpch_2016_2017	tfpch_2017_2018	tfpch_mean
Arithmetic mean	1.04.688	1.00.658	.99.263
Std. deviation	0.374496	0.379603	0.234574
Minimum	0.213	0.270	0.501
Maximum	3.424	3.627	2.420

Ν: 155, Valid: 155, Missing: 0

In overall productivity change, Health Centre 22 has the maximum improvement, while Health Centre 120 has the maximum decline.

Table 4 shows the geometric mean of total productivity for the 155 health centres over 2016–2017 and 2017–2018 and the geometric mean values of efficiency, divided into technical and scale efficiency, and technology. The table also presents the number of health centres whose productivity increased, decreased, or remained constant over the two periods.

**Table 4:** Summary scores and number of health centres that progressed, regressed, or remained constant

Geometric Mean	2016–2017	2017–2018	Total (2016–2018)
effch	0.994	1.007	1.000
techch	0.997	0.941	0.969
pech	0.980	1.006	0.993
sech	1.014	1.001	1.008
tfpch	0.991	0.948	0.969
DMU’s that progressed (tfpch > 1)	73 (47.09%)	72 (46.45%)	61 (39.35%)
DMU’s that regressed (tfpch < 1)	82 (52.9%)	83 (53.54%)	94 (60.64%)
DMU’s remained constant (tfpch = 1)	0	0	0

In the first period (2016–2017), 73 health centres achieved progress in their total productivity, while 82 had lower productivity levels. In the second period, 72 health centres achieved progress in their total productivity, while 83 had lower productivity levels. Overall productivity for both periods showed an increase for 61 health centres and a decrease for 94 health centres.

After monitoring the productivity change of each health centre, the factor that contributed mainly to the change was explained (Table 5).

**Table 5:** Summary results, number of progressed, regressed or remained constant Health Centers

PERIOD	2016–2017	2017–2018	Total (2016–2018)
Change into effch			
Prog. (effch > 1)	48 (30.96%)	43 (27.74%)	51 (32.90%)
Reg. (effch < 1)	43 (27.74%)	44 (28.38%)	46 (29.67%)
Constant (effch = 1)	64 (41.29%)	68 (43.87%)	58 (37.41%)
Change into techch			
Prog. (techch > 1)	74 (47.74%)	62 (40%)	54 (34.83%)
Reg. (techch < 1)	81 (52.25%)	93 (60%)	101 (65.16%)
Constant (techch = 1)	0	0	0
Change into tfpch			
Prog. (tfpch > 1)	73 (47.09%)	72 (46.45%)	61 (39.35%)
Reg. (tfpch < 1)	82 (52.9%)	83 (53.54%)	94 (60.64%)
Constant (tfpch = 1)	0	0	0

In the first period (2016–2017), 48 health centres showed improvement in their technical efficiency, 43 showed a decline in their technical efficiency, and 64 achieved the same levels of technical efficiency. In addition, 74 health centres improved their technology, while 81 showed a decline in their technology. In the second period (2017–2018), 43 health centres showed improvement in their technical efficiency, 44 showed a decline in their technical efficiency, and 68 achieved the same levels of technical efficiency. In addition, 62 health centres showed technology improvement, while 93 health centres showed a decline in their technology for the second period.

The analysis of the mean values showed a decrease of 0.9% in the overall productivity factor from 2016 to 2017 and a decrease of 5.2% from 2017 to 2018. The overall decrease in the productivity of the 155 health centres was 3.1%.

To examine the change in total productivity, the difference between effch and techch for each health centre is calculated. Positive values for the difference mean that the change in productivity is mainly due to a change in technical efficiency, while negative values for the difference mean that productivity change is mainly due to a change in technology for a given health centre [42].

Moreover, since effch = pech*sech, if pech > sech is true, then the change in technical efficiency is mainly due to pure technical efficiency progress or regression. In contrast if it is false, then the change in technical efficiency is mainly due to the change in scale efficiency for a given health centre.

From 2016 to 2018, 59 health centres changed their productivity mainly due to technological change, 91 mainly due to technical efficiency change, while one health centre showed regression to its total productivity due to equal regression of its technical efficiency and technology.

**Fig. 1:**
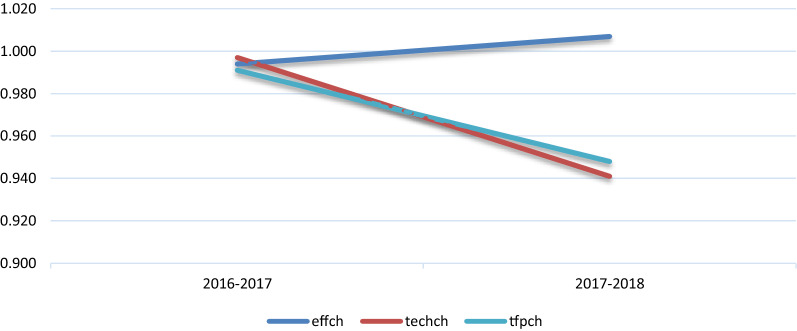
Tfpch, techch and effch over time (Geometric Mean Values)

It should be stated that the majority of health centres changed their productivity over time due to technical efficiency change and that the health centres with high volatility in their productivity had high volatility in their technology, which shows that the largest changes in productivity were mostly due to technology change. This explains why, in Fig. 1, when the mean productivity and the mean technology change over time, the changes through the 2 periods show a similar movement.

### Model validation

To test the internal and external validity of the MPI DEA input-oriented model, the Spearman’s rank correlation test was performed. Internal validity compares whether there are differences in the overall productivity of health centres when different inputs and outputs are used. In contrast, external validity compares whether there is consistency based on data from different periods. DEA is a nonparametric method, so it is not possible to directly compare productivity and efficiency by different models. However, the comparison of efficiencies and productivity can be applied through nonparametric correlation tests [43–46].

The total productivities of health centres were calculated using different models with different inputs and outputs. The models are presented in Table 6, which shows the different inputs and outputs used to calculate each model’s efficiencies, as well as the total productivities of the health centres by each model.

**Table 6:** Different models for internal validity check

Models/Variables	Out1	Out2	Out3	Out4	Out5	Out6	Out7	Out8	Out9	Out10	Out11	Out12	Inp1	Inp2	Inp3	Inp4
M0	Χ	Χ	Χ	Χ	Χ	Χ	Χ	Χ	Χ	Χ	Χ	Χ	Χ	Χ	Χ	Χ
M1	Χ	Χ	Χ	Χ	Χ	Χ	Χ	Χ	Χ	Χ	Χ	Χ		Χ	Χ	Χ
M2	Χ	Χ	Χ			Χ	Χ	Χ	Χ	Χ	Χ	Χ	Χ	Χ	Χ	Χ
M3	Χ	Χ		Χ	Χ	Χ	Χ		Χ		Χ	Χ	Χ		Χ	

The first model (MO) considers all inputs and outputs, the second model (M1) excludes the input variable “Number of Managers”, the third model (M2) excludes the output variables “Chronic Disease Cases” and “Emergencies”, while the fourth model (M3) excludes the input variables “Number of Managers” and “Number of nonmedical staff” and the output variables “Test Mantoux”, “Dental Procedures” and “Transcriptions”.

After estimating the total productivities of the 155 health centres, under the input-oriented MPI DEA, the Spearman’s rank correlation coefficients were calculated for the four different models (Table 7).

**Table 7:** Spearman rank correlation (model internal validity test—total productivity)

	tfpch (M0)	tfpch (M1)	tfpch (M2)	tfpch (M3)
tfpch (M0)	1.000	0.896^**^	0.951^**^	0.766^**^
tfpch (M1)	0.896^**^	1.000	0.862^**^	0.814^**^
tfpch (M2)	0.951^**^	0.862^**^	1.000	0.708^**^
tfpch (M3)	0.766^**^	0.814^**^	0.708^**^	1.000

^**^Correlation is significant at the 0.01 level (2-tailed). N: 155

The Spearman’s rank correlation tests for internal validity show statistically significant correlations between the different model specifications.

To subject the analysis to an external validity test, the Spearman’s rank correlation test between the calculated total productivities over the two periods was performed (Table 8).

**Table 8:** Spearman rank correlation (model external validity test—productivity of each period)

	tfpch (period1)	tfpch (period2)
tfpch (period1)	1.000	− 0.250**
tfpch (period2)	− 0.250**	1.000

^**^Correlation is significant at the 0.01 level (2-tailed). N: 155

The Spearman’s rank correlation tests for external validity show a low but statistically significant correlation between periods.

The Spearman’s rank coefficients re-ensure strong external and internal validity for the model.

## Discussion

The dataset was provided by the Ministry of Health and covered the years 2016, 2017 and 2018. Attempts were made to collect data for all 207 health centres of Greece, as well as financial data for the health centres, which would have been used as inputs for this paper and would have contributed to the evaluation of the total productivities and efficiencies of the health centres. The financial resources of each health centre constitute a vital parameter in the evaluation of their efficiencies and productivities, since financial resources determine health centres’ resources for operating and providing health services. Unfortunately, data on costs and expenditures for the health centres were missing. Still, since that health centres are labour-intensive units, the total staff employed and occupied was used as inputs for the estimation of the total productivities and efficiencies. It is recommended that the Greek government start gathering financial data to estimate the total productivity and efficiency of primary health care [27]. In addition, 52 health centres were excluded from this paper due to lack of data, mainly due to the change in the system that the data were collected in 2016.

## Conclusion

In this paper, the nonparametric DEA method, along with the MPI, was used to estimate the productivity change during the period 2016–2018. Moreover, after the estimation of productivity of each health centre for the years 2016, 2017 and 2018, as well as the change in productivity of each health centre over time, the factors that contributed most were estimated, shedding light on the changes in technical efficiency and technology for the health centres during 2016–2018.

Meaningful results were extracted by calculating the number of health centres that improved their productivity, regressed in productivity or remained constant through 2016–2018. Additionally, productivity evaluation is critical since it provides important information about the viability and efficiency of each health centre and shows the overall growth and progress of primary health care [11].

In a previous paper for health centres in Greece for 2018, a Tobit regression analysis was employed to investigate if there was a correlation between their productivity levels and the health region to which they belonged: “since they operate in district areas with differences and peculiarities in many aspects, such as concentration of people in their region, an environmental factor which may affect the health of the overall population, availability to employ specialized workforce and hospitals nearby health centres that it may affect” [12, 28].The study showed that the efficiency of the health centres was not affected by the health region to which they belong [19]. Moreover, the average efficiency of the 198 Health Centers for 2018 was 0.916, but the study did not consider panel data. Extensive research should be conducted to investigate exogenous factors that may affect the efficiency and productivity of health centres such as demographic, socioeconomic, and community criteria, as well as environmental factors. Moreover, meaningful results can be extracted by evaluating the efficiency and productivity of TOMY after the first years of their operation.

Greece’s economic crisis affected the country’s primary health care, leading to a decline in the overall efficiencies and productivity of health centres. The first years of the reform did not have the expected results in primary health care. The reform fell into a lack of planning and physical resources, which, along with the missing guidelines, the absence of organization and referral systems led to people's dissatisfaction. However, the gradual reshaping of the primary health care system and the initiation of the population in primary health care services are expected to give encouraging results in the near future. The reforms created a framework in which significant steps can be made to improve the primary health care system.

This paper may contribute to improving health centres’ efficiency and productivity. Furthermore, valuable results can be extracted for the National Health Care System to match available resources depending on each centre’s needs, as well as for manager planners and stakeholders in primary health care.

It is globally accepted that health systems with strong people-centred, continuous, comprehensive and coordinated PHC are more efficient [8]. To achieve better results, it is strongly recommended for the government and stakeholders to better distribute the personnel occupied in health centres to introduce continuous professional development among the specialized workforce. In addition, health centres that face inefficiencies should take as an example from the efficient centres. Achieving the above will lead people to embrace the primary health care system and utilize health centres and TOMY services, laying the foundation for the success of the reform.

The estimation of total productivities and efficiencies of health centres were calculated using DEAP version 2.1 for Windows by Coelli (1996). Statistics were performed by using the IBM SPSS program.

## Supplementary Information

**Additional file 1**: Includes detailed table with Descriptive Statistics of inputs and outputs for the years 2016, 2017 and 2018.

**Additional file 2**: Includes detailed table with Malmquist Productivity Index results for periods 2016-2017, 2017-2018 and overall results for both periods.

## Data Availability

The data that support the findings of this study are available from Bi-health (Ministry of Health) but restrictions apply to the availability of these data, which were used under license for the current study, and so are not publicly available. Data are however available from the authors upon reasonable request and with permission of Ministry of Health.

## Abbreviations

WHO: World Health Organization
OECD: Organization for Economic Co-operation and Development
PEDY: National Primary Health Care Network
ESY: Regional Health Care Authorities
TOMY: Local health unit
DEA: Data envelopment analysis
DMU: Decision making unit
CRS: Constant return to scale
VRS: Variable return to scale
MPI: Malmquist productivity index
Effch: Efficiency change
Techch: Technology change
Pech: Pure efficiency change
Sech: Scale efficiency change
Tfpch: Total factor productivity change
