# Supplementary material for: Total productivity change of Health Centers in Greece in 2016–2018: a Malmquist index data envelopment analysis application for the primary health system of Greece
Source: Cost Eff Resour Alloc. 2021 Nov 2;19:72. doi: 10.1186/s12962-021-00326-z (PMC8561945; doi:10.1186/s12962-021-00326-z)
Supplement: Supplementary file 1 — Additional file 1: Includes detailed table with Descriptive Statistics of inputs and outputs for the years 2016, 2017 and 2018. [file 12962_2021_326_MOESM1_ESM.docx]

| **Descriptive Statistics of inputs and outputs for the years 2016, 2017 and 2018.** | | | | |
| --- | --- | --- | --- | --- |
| **Variables** | **Statistics** | **2016** | **2017** | **2018** |
| **Nursing Operations** | *Mean* | 3666,52 | 4344,50 | 4521,98 |
|  | *St. Dev.* | 4641,111 | 4820,566 | 4492,954 |
|  | *Min* | 0 | 0 | 0 |
|  | *Max* | 36235 | 30692 | 28457 |
| **Microsurgeries** | *Mean* | 354,90 | 713,39 | 357,28 |
|  | *St. Dev.* | 665,668 | 4243,418 | 502,986 |
|  | *Min* | 0 | 0 | 0 |
|  | *Max* | 6164 | 52638 | 2968 |
| **Dental Procedures** | *Mean* | 1123,79 | 1244,55 | 1206,97 |
|  | *St. Dev.* | 1552,365 | 1791,178 | 1754,460 |
|  | *Min* | 0 | 0 | 0 |
|  | *Max* | 9583 | 13647 | 11022 |
| **Chronic Disease Cases** | *Mean* | 4134,47 | 4492,99 | 5032,21 |
|  | *St. Dev.* | 6687,206 | 7143,300 | 8733,564 |
|  | *Min* | 0 | 0 | 0 |
|  | *Max* | 41851 | 34717 | 47697 |
| **Emergency cases** | *Mean* | 1588,64 | 1642,44 | 1761,20 |
|  | *St. Dev.* | 2140,112 | 2130,753 | 2193,249 |
|  | *Min* | 0 | 0 | 0 |
|  | *Max* | 21042 | 20155 | 20131 |
| **Regular Incidents** | *Mean* | 13367,20 | 13864,37 | 13704,56 |
|  | *St. Dev.* | 10112,258 | 10358,008 | 10480,201 |
|  | *Min* | 0 | 0 | 0 |
|  | *Max* | 63247 | 67078 | 69195 |
| **Urgent Incidents** | *Mean* | 8537,79 | 9063,77 | 9787,39 |
|  | *St. Dev.* | 6914,711 | 7332,581 | 8632,499 |
|  | *Min* | 851 | 979 | 0 |
|  | *Max* | 33773 | 38780 | 46759 |
| **Transcriptions** | *Mean* | 12216,85 | 11829,95 | 10991,45 |
|  | *St. Dev.* | 9735,944 | 9314,838 | 9179,916 |
|  | *Min* | 0 | 0 | 0 |
|  | *Max* | 62545 | 65522 | 56313 |
| **Bio-pathological and laboratory exams** | *Mean* | 16040,90 | 14265,41 | 16084,43 |
|  | *St. Dev.* | 21139,547 | 22556,482 | 24890,277 |
|  | *Min* | 0 | 0 | 0 |
|  | *Max* | 124501 | 154180 | 160149 |
| **Test Mantoux** | *Mean* | 41,92 | 106,01 | 46,53 |
|  | *St. Dev.* | 72,196 | 190,534 | 93,678 |
|  | *Min* | 0 | 0 | 0 |
|  | *Max* | 419 | 1608 | 640 |
| **Vaccinations applied for adults** | *Mean* | 469,72 | 474,58 | 552,32 |
|  | *St. Dev.* | 583,374 | 632,540 | 675,561 |
|  | *Min* | 0 | 0 | 0 |
|  | *Max* | 4109 | 4409 | 3948 |
| **Vaccinations applied for kids and teenagers** | *Mean* | 450,11 | 536,06 | 571,86 |
|  | *St. Dev.* | 540,705 | 641,459 | 658,154 |
|  | *Min* | 0 | 0 | 0 |
|  | *Max* | 2861 | 2987 | 2754 |
| **Number of Managers** | *Mean* | 2,43 | 2,58 | 2,70 |
|  | *St. Dev.* | 1,893 | 1,713 | 1,820 |
|  | *Min* | 1 | 1 | 1 |
|  | *Max* | 12 | 10 | 12 |
| **Number of Doctors** | *Mean* | 8,92 | 9,45 | 10,03 |
|  | *St. Dev.* | 6,066 | 6,514 | 7,270 |
|  | *Min* | 1 | 1 | 2 |
|  | *Max* | 35 | 34 | 37 |
| **Number of Nursing staff** | *Mean* | 15,64 | 16,32 | 16,79 |
|  | *St. Dev.* | 10,301 | 9,779 | 9,890 |
|  | *Min* | 2 | 2 | 1 |
|  | *Max* | 70 | 46 | 48 |
| **Number of Non-Medical staff** | *Mean* | 6,97 | 7,17 | 8,01 |
|  | *St. Dev.* | 4,237 | 4,408 | 4,721 |
|  | *Min* | 1 | 1 | 1 |
|  | *Max* | 34 | 32 | 30 |
| **Valid N (listwise)** | *155* |  |  |  |
