# Supplementary material for: Total productivity change of Health Centers in Greece in 2016–2018: a Malmquist index data envelopment analysis application for the primary health system of Greece
Source: Cost Eff Resour Alloc. 2021 Nov 2;19:72. doi: 10.1186/s12962-021-00326-z (PMC8561945; doi:10.1186/s12962-021-00326-z)
Supplement: Supplementary file 2 — Additional file 2: Includes detailed table with Malmquist Productivity Index results for periods 2016-2017, 2017-2018 and overall results for both periods. [file 12962_2021_326_MOESM2_ESM.docx]

| **Malmquist Productivity Index results for periods 2016-2017, 2017-2018 and overall results for both periods** | | | | | | | | | | | | | | | |
| --- | --- | --- | --- | --- | --- | --- | --- | --- | --- | --- | --- | --- | --- | --- | --- |
| ***a/a*** | ***Time period 2016-2017*** | | | | | ***Time period 2017-2018*** | | | | | ***Overall Results 2016-2018*** | | | | |
| ***DMU’s*** | ***effch*** | ***techch*** | ***pech*** | ***sech*** | ***tfpch*** | ***effch*** | ***techch*** | ***pech*** | ***sech*** | ***tfpch*** | ***effch*** | ***techch*** | ***pech*** | ***sech*** | ***tfpch*** |
| 1 | 1,078 | 1,094 | 1,000 | 1,078 | 1,179 | 1,000 | 0,900 | 1,000 | 1,000 | 0,900 | 1,038 | 0,992 | 1,000 | 1,038 | 1,030 |
| 2 | 1,000 | 1,032 | 1,000 | 1,000 | 1,032 | 0,987 | 0,856 | 0,998 | 0,989 | 0,846 | 0,994 | 0,940 | 0,999 | 0,994 | 0,934 |
| 3 | 0,907 | 1,094 | 0,936 | 0,968 | 0,992 | 1,103 | 0,842 | 1,068 | 1,033 | 0,929 | 1,000 | 0,960 | 1,000 | 1,000 | 0,960 |
| 4 | 1,000 | 1,354 | 1,000 | 1,000 | 1,354 | 1,000 | 0,611 | 1,000 | 1,000 | 0,611 | 1,000 | 0,909 | 1,000 | 1,000 | 0,909 |
| 5 | 1,055 | 0,980 | 0,977 | 1,080 | 1,034 | 0,952 | 0,947 | 0,973 | 0,978 | 0,901 | 1,002 | 0,963 | 0,975 | 1,028 | 0,966 |
| 6 | 1,050 | 0,969 | 1,000 | 1,050 | 1,018 | 0,940 | 0,962 | 0,759 | 1,239 | 0,904 | 0,994 | 0,965 | 0,871 | 1,141 | 0,959 |
| 7 | 1,000 | 2,413 | 1,000 | 1,000 | 2,413 | 1,000 | 0,270 | 1,000 | 1,000 | 0,270 | 1,000 | 0,807 | 1,000 | 1,000 | 0,807 |
| 8 | 1,089 | 1,018 | 1,000 | 1,089 | 1,109 | 0,929 | 0,927 | 0,895 | 1,038 | 0,861 | 1,005 | 0,972 | 0,946 | 1,063 | 0,977 |
| 9 | 1,000 | 1,071 | 1,000 | 1,000 | 1,071 | 1,000 | 1,014 | 1,000 | 1,000 | 1,014 | 1,000 | 1,042 | 1,000 | 1,000 | 1,042 |
| 10 | 1,000 | 0,818 | 1,000 | 1,000 | 0,818 | 0,730 | 0,870 | 0,730 | 1,000 | 0,635 | 0,854 | 0,843 | 0,854 | 1,000 | 0,720 |
| 11 | 0,689 | 1,088 | 1,048 | 0,657 | 0,750 | 1,302 | 0,926 | 1,029 | 1,266 | 1,205 | 0,947 | 1,004 | 1,038 | 0,912 | 0,951 |
| 12 | 0,903 | 1,119 | 0,892 | 1,013 | 1,010 | 0,901 | 0,884 | 0,944 | 0,954 | 0,796 | 0,902 | 0,994 | 0,917 | 0,983 | 0,897 |
| 13 | 1,000 | 1,157 | 1,000 | 1,000 | 1,157 | 1,000 | 0,786 | 1,000 | 1,000 | 0,786 | 1,000 | 0,953 | 1,000 | 1,000 | 0,953 |
| 14 | 1,000 | 1,149 | 1,000 | 1,000 | 1,149 | 1,000 | 0,470 | 1,000 | 1,000 | 0,470 | 1,000 | 0,735 | 1,000 | 1,000 | 0,735 |
| 15 | 1,000 | 0,875 | 1,000 | 1,000 | 0,875 | 0,551 | 0,595 | 0,693 | 0,796 | 0,328 | 0,742 | 0,722 | 0,832 | 0,892 | 0,536 |
| 16 | 1,111 | 1,438 | 1,084 | 1,025 | 1,598 | 0,900 | 0,577 | 0,921 | 0,978 | 0,519 | 1,000 | 0,911 | 0,999 | 1,001 | 0,911 |
| 17 | 1,118 | 0,821 | 0,966 | 1,158 | 0,918 | 1,132 | 1,037 | 1,086 | 1,042 | 1,174 | 1,125 | 0,923 | 1,024 | 1,099 | 1,038 |
| 18 | 0,742 | 0,956 | 0,810 | 0,916 | 0,709 | 0,761 | 1,219 | 1,018 | 0,747 | 0,927 | 0,751 | 1,080 | 0,908 | 0,827 | 0,811 |
| 19 | 1,000 | 1,186 | 1,000 | 1,000 | 1,186 | 1,000 | 1,014 | 1,000 | 1,000 | 1,014 | 1,000 | 1,096 | 1,000 | 1,000 | 1,096 |
| 20 | 0,651 | 1,712 | 0,662 | 0,984 | 1,115 | 1,347 | 0,725 | 1,511 | 0,891 | 0,977 | 0,937 | 1,114 | 1,000 | 0,937 | 1,043 |
| 21 | 1,000 | 0,770 | 1,000 | 1,000 | 0,770 | 1,000 | 0,945 | 1,000 | 1,000 | 0,945 | 1,000 | 0,853 | 1,000 | 1,000 | 0,853 |
| 22 | 1,407 | 1,148 | 1,000 | 1,407 | 1,614 | 3,430 | 1,057 | 1,000 | 3,430 | 3,627 | 2,197 | 1,102 | 1,000 | 2,197 | 2,420 |
| 23 | 1,000 | 1,224 | 1,000 | 1,000 | 1,224 | 1,000 | 0,704 | 1,000 | 1,000 | 0,704 | 1,000 | 0,928 | 1,000 | 1,000 | 0,928 |
| 24 | 1,042 | 1,049 | 1,039 | 1,003 | 1,092 | 1,000 | 1,148 | 1,000 | 1,000 | 1,148 | 1,021 | 1,097 | 1,019 | 1,002 | 1,120 |
| 25 | 1,106 | 0,981 | 1,071 | 1,033 | 1,085 | 1,000 | 1,280 | 1,000 | 1,000 | 1,280 | 1,052 | 1,121 | 1,035 | 1,016 | 1,179 |
| 26 | 1,000 | 0,896 | 1,000 | 1,000 | 0,896 | 0,584 | 1,005 | 0,588 | 0,992 | 0,586 | 0,764 | 0,949 | 0,767 | 0,996 | 0,725 |
| 27 | 0,921 | 1,075 | 1,000 | 0,921 | 0,989 | 0,882 | 0,993 | 0,813 | 1,085 | 0,876 | 0,901 | 1,033 | 0,902 | 0,999 | 0,931 |
| 28 | 1,000 | 0,808 | 1,000 | 1,000 | 0,808 | 1,000 | 1,098 | 1,000 | 1,000 | 1,098 | 1,000 | 0,942 | 1,000 | 1,000 | 0,942 |
| 29 | 1,050 | 1,165 | 1,009 | 1,040 | 1,223 | 0,876 | 0,877 | 0,849 | 1,032 | 0,769 | 0,959 | 1,011 | 0,926 | 1,036 | 0,970 |
| 30 | 1,000 | 0,971 | 1,000 | 1,000 | 0,971 | 1,000 | 0,791 | 1,000 | 1,000 | 0,791 | 1,000 | 0,877 | 1,000 | 1,000 | 0,877 |
| 31 | 1,063 | 0,936 | 1,024 | 1,038 | 0,995 | 1,000 | 0,982 | 1,000 | 1,000 | 0,982 | 1,031 | 0,959 | 1,012 | 1,019 | 0,988 |
| 32 | 0,739 | 1,140 | 0,474 | 1,559 | 0,842 | 1,611 | 0,754 | 2,110 | 0,763 | 1,215 | 1,091 | 0,927 | 1,000 | 1,091 | 1,012 |
| 33 | 1,042 | 0,954 | 1,027 | 1,014 | 0,993 | 1,389 | 0,902 | 1,127 | 1,233 | 1,253 | 1,203 | 0,927 | 1,076 | 1,118 | 1,116 |
| 34 | 1,000 | 1,201 | 1,000 | 1,000 | 1,201 | 1,000 | 0,526 | 1,000 | 1,000 | 0,526 | 1,000 | 0,795 | 1,000 | 1,000 | 0,795 |
| 35 | 1,021 | 0,997 | 0,817 | 1,250 | 1,018 | 1,138 | 0,993 | 1,106 | 1,029 | 1,130 | 1,078 | 0,995 | 0,950 | 1,134 | 1,072 |
| 36 | 1,000 | 0,906 | 1,000 | 1,000 | 0,906 | 1,000 | 1,057 | 1,000 | 1,000 | 1,057 | 1,000 | 0,978 | 1,000 | 1,000 | 0,978 |
| 37 | 0,766 | 0,900 | 1,000 | 0,766 | 0,690 | 0,969 | 0,932 | 1,000 | 0,969 | 0,903 | 0,862 | 0,916 | 1,000 | 0,862 | 0,789 |
| 38 | 1,000 | 0,910 | 1,000 | 1,000 | 0,910 | 1,000 | 0,957 | 1,000 | 1,000 | 0,957 | 1,000 | 0,933 | 1,000 | 1,000 | 0,933 |
| 39 | 1,000 | 0,904 | 1,000 | 1,000 | 0,904 | 1,000 | 1,001 | 1,000 | 1,000 | 1,001 | 1,000 | 0,951 | 1,000 | 1,000 | 0,951 |
| 40 | 1,000 | 0,750 | 1,000 | 1,000 | 0,750 | 1,000 | 0,888 | 1,000 | 1,000 | 0,888 | 1,000 | 0,816 | 1,000 | 1,000 | 0,816 |
| 41 | 0,570 | 0,890 | 0,518 | 1,101 | 0,507 | 1,297 | 0,927 | 1,354 | 0,958 | 1,203 | 0,860 | 0,908 | 0,837 | 1,027 | 0,781 |
| 42 | 1,000 | 1,021 | 1,000 | 1,000 | 1,021 | 1,000 | 0,715 | 1,000 | 1,000 | 0,715 | 1,000 | 0,854 | 1,000 | 1,000 | 0,854 |
| 43 | 1,010 | 0,911 | 0,965 | 1,046 | 0,920 | 1,044 | 1,024 | 3,258 | 0,320 | 1,069 | 1,027 | 0,966 | 1,773 | 0,579 | 0,992 |
| 44 | 0,655 | 0,638 | 0,768 | 0,853 | 0,418 | 1,526 | 1,142 | 1,303 | 1,172 | 1,743 | 1,000 | 0,854 | 1,000 | 1,000 | 0,854 |
| 45 | 1,000 | 0,941 | 1,000 | 1,000 | 0,941 | 1,000 | 0,913 | 1,000 | 1,000 | 0,913 | 1,000 | 0,927 | 1,000 | 1,000 | 0,927 |
| 46 | 0,846 | 0,895 | 1,000 | 0,846 | 0,757 | 0,985 | 1,065 | 1,000 | 0,985 | 1,049 | 0,913 | 0,976 | 1,000 | 0,913 | 0,891 |
| 47 | 1,000 | 0,853 | 1,000 | 1,000 | 0,853 | 0,948 | 0,929 | 1,000 | 0,948 | 0,881 | 0,974 | 0,890 | 1,000 | 0,974 | 0,866 |
| 48 | 1,000 | 0,987 | 1,000 | 1,000 | 0,987 | 1,000 | 1,164 | 1,000 | 1,000 | 1,164 | 1,000 | 1,072 | 1,000 | 1,000 | 1,072 |
| 49 | 1,000 | 0,975 | 1,000 | 1,000 | 0,975 | 0,751 | 0,852 | 0,773 | 0,972 | 0,640 | 0,867 | 0,911 | 0,879 | 0,986 | 0,790 |
| 50 | 1,178 | 0,986 | 1,130 | 1,042 | 1,162 | 1,000 | 0,806 | 1,000 | 1,000 | 0,806 | 1,085 | 0,891 | 1,063 | 1,021 | 0,968 |
| 51 | 1,000 | 0,879 | 1,000 | 1,000 | 0,879 | 1,000 | 0,900 | 1,000 | 1,000 | 0,900 | 1,000 | 0,890 | 1,000 | 1,000 | 0,890 |
| 52 | 1,000 | 0,691 | 1,000 | 1,000 | 0,691 | 1,000 | 1,147 | 1,000 | 1,000 | 1,147 | 1,000 | 0,890 | 1,000 | 1,000 | 0,890 |
| 53 | 0,647 | 0,826 | 0,553 | 1,169 | 0,534 | 2,048 | 0,867 | 1,708 | 1,199 | 1,776 | 1,151 | 0,846 | 0,972 | 1,184 | 0,974 |
| 54 | 1,786 | 1,263 | 1,371 | 1,303 | 2,256 | 1,000 | 1,589 | 1,000 | 1,000 | 1,589 | 1,336 | 1,417 | 1,171 | 1,141 | 1,894 |
| 55 | 1,000 | 0,947 | 1,000 | 1,000 | 0,947 | 0,885 | 1,039 | 0,945 | 0,936 | 0,919 | 0,941 | 0,992 | 0,972 | 0,967 | 0,933 |
| 56 | 1,033 | 1,061 | 1,000 | 1,033 | 1,096 | 0,757 | 1,089 | 0,735 | 1,030 | 0,824 | 0,884 | 1,075 | 0,857 | 1,031 | 0,951 |
| 57 | 1,104 | 1,376 | 1,018 | 1,084 | 1,519 | 1,000 | 0,780 | 1,000 | 1,000 | 0,780 | 1,051 | 1,036 | 1,009 | 1,041 | 1,088 |
| 58 | 1,000 | 1,059 | 1,000 | 1,000 | 1,059 | 1,000 | 0,835 | 1,000 | 1,000 | 0,835 | 1,000 | 0,941 | 1,000 | 1,000 | 0,941 |
| 59 | 1,000 | 0,787 | 1,000 | 1,000 | 0,787 | 1,000 | 1,036 | 1,000 | 1,000 | 1,036 | 1,000 | 0,903 | 1,000 | 1,000 | 0,903 |
| 60 | 1,000 | 0,868 | 1,000 | 1,000 | 0,868 | 1,000 | 1,332 | 1,000 | 1,000 | 1,332 | 1,000 | 1,075 | 1,000 | 1,000 | 1,075 |
| 61 | 1,000 | 0,998 | 1,000 | 1,000 | 0,998 | 1,000 | 0,761 | 1,000 | 1,000 | 0,761 | 1,000 | 0,871 | 1,000 | 1,000 | 0,871 |
| 62 | 1,628 | 0,760 | 1,000 | 1,628 | 1,237 | 1,000 | 1,241 | 1,000 | 1,000 | 1,241 | 1,276 | 0,971 | 1,000 | 1,276 | 1,239 |
| 63 | 1,565 | 0,633 | 1,000 | 1,565 | 0,991 | 1,069 | 1,182 | 1,000 | 1,069 | 1,263 | 1,293 | 0,865 | 1,000 | 1,293 | 1,119 |
| 64 | 1,783 | 0,902 | 1,000 | 1,783 | 1,609 | 0,769 | 1,055 | 0,723 | 1,064 | 0,812 | 1,171 | 0,976 | 0,851 | 1,377 | 1,143 |
| 65 | 1,000 | 0,893 | 1,000 | 1,000 | 0,893 | 1,000 | 0,999 | 1,000 | 1,000 | 0,999 | 1,000 | 0,944 | 1,000 | 1,000 | 0,944 |
| 66 | 0,771 | 0,820 | 0,699 | 1,103 | 0,633 | 1,311 | 1,083 | 1,430 | 0,917 | 1,420 | 1,006 | 0,943 | 1,000 | 1,006 | 0,948 |
| 67 | 0,688 | 0,953 | 0,555 | 1,241 | 0,656 | 1,426 | 0,966 | 1,803 | 0,791 | 1,377 | 0,991 | 0,959 | 1,000 | 0,991 | 0,950 |
| 68 | 0,931 | 0,821 | 0,883 | 1,054 | 0,765 | 0,661 | 0,750 | 0,661 | 1,000 | 0,495 | 0,785 | 0,785 | 0,764 | 1,027 | 0,616 |
| 69 | 1,003 | 0,826 | 1,029 | 0,975 | 0,828 | 1,225 | 1,098 | 1,178 | 1,040 | 1,345 | 1,109 | 0,952 | 1,101 | 1,007 | 1,056 |
| 70 | 1,182 | 0,819 | 1,000 | 1,182 | 0,969 | 0,323 | 1,018 | 0,333 | 0,970 | 0,329 | 0,618 | 0,913 | 0,577 | 1,071 | 0,565 |
| 71 | 0,713 | 0,826 | 0,758 | 0,941 | 0,589 | 0,971 | 1,120 | 0,940 | 1,033 | 1,087 | 0,832 | 0,962 | 0,844 | 0,986 | 0,800 |
| 72 | 0,870 | 0,843 | 1,000 | 0,870 | 0,734 | 1,149 | 1,200 | 1,000 | 1,149 | 1,379 | 1,000 | 1,006 | 1,000 | 1,000 | 1,006 |
| 73 | 1,135 | 0,858 | 1,069 | 1,061 | 0,973 | 0,863 | 0,999 | 0,875 | 0,986 | 0,862 | 0,990 | 0,926 | 0,967 | 1,023 | 0,916 |
| 74 | 1,000 | 1,116 | 1,000 | 1,000 | 1,116 | 1,000 | 0,860 | 1,000 | 1,000 | 0,860 | 1,000 | 0,980 | 1,000 | 1,000 | 0,980 |
| 75 | 3,256 | 1,051 | 1,691 | 1,925 | 3,424 | 0,923 | 1,095 | 0,994 | 0,928 | 1,010 | 1,733 | 1,073 | 1,297 | 1,337 | 1,859 |
| 76 | 0,983 | 1,016 | 0,981 | 1,002 | 0,999 | 1,171 | 0,872 | 1,134 | 1,033 | 1,021 | 1,073 | 0,941 | 1,055 | 1,017 | 1,010 |
| 77 | 0,945 | 1,075 | 1,000 | 0,945 | 1,016 | 1,187 | 0,843 | 1,000 | 1,187 | 1,001 | 1,059 | 0,952 | 1,000 | 1,059 | 1,008 |
| 78 | 0,946 | 0,760 | 0,992 | 0,953 | 0,719 | 1,057 | 1,011 | 1,008 | 1,049 | 1,069 | 1,000 | 0,877 | 1,000 | 1,000 | 0,877 |
| 79 | 1,000 | 0,887 | 1,000 | 1,000 | 0,887 | 1,000 | 1,012 | 1,000 | 1,000 | 1,012 | 1,000 | 0,947 | 1,000 | 1,000 | 0,947 |
| 80 | 0,860 | 1,105 | 1,363 | 0,631 | 0,950 | 1,010 | 1,013 | 1,000 | 1,010 | 1,023 | 0,932 | 1,058 | 1,168 | 0,798 | 0,986 |
| 81 | 1,354 | 1,030 | 1,000 | 1,354 | 1,395 | 1,344 | 0,944 | 1,000 | 1,344 | 1,268 | 1,349 | 0,986 | 1,000 | 1,349 | 1,330 |
| 82 | 0,899 | 1,039 | 0,914 | 0,984 | 0,934 | 1,019 | 0,990 | 1,071 | 0,951 | 1,009 | 0,957 | 1,014 | 0,989 | 0,968 | 0,971 |
| 83 | 1,377 | 1,048 | 1,321 | 1,042 | 1,443 | 1,000 | 0,957 | 1,000 | 1,000 | 0,957 | 1,173 | 1,001 | 1,149 | 1,021 | 1,175 |
| 84 | 1,344 | 1,085 | 1,253 | 1,073 | 1,459 | 1,115 | 0,966 | 1,098 | 1,015 | 1,077 | 1,224 | 1,024 | 1,173 | 1,044 | 1,253 |
| 85 | 0,935 | 0,751 | 0,979 | 0,955 | 0,702 | 0,662 | 1,178 | 0,692 | 0,957 | 0,780 | 0,787 | 0,940 | 0,823 | 0,956 | 0,740 |
| 86 | 1,000 | 0,746 | 1,000 | 1,000 | 0,746 | 1,000 | 2,155 | 1,000 | 1,000 | 2,155 | 1,000 | 1,268 | 1,000 | 1,000 | 1,268 |
| 87 | 1,000 | 1,190 | 1,000 | 1,000 | 1,190 | 0,490 | 0,762 | 0,491 | 1,000 | 0,374 | 0,700 | 0,952 | 0,700 | 1,000 | 0,667 |
| 88 | 1,049 | 1,039 | 1,001 | 1,048 | 1,090 | 0,882 | 0,837 | 0,976 | 0,903 | 0,738 | 0,962 | 0,933 | 0,989 | 0,973 | 0,897 |
| 89 | 1,000 | 0,908 | 1,000 | 1,000 | 0,908 | 1,000 | 0,924 | 1,000 | 1,000 | 0,924 | 1,000 | 0,916 | 1,000 | 1,000 | 0,916 |
| 90 | 0,745 | 0,853 | 1,000 | 0,745 | 0,636 | 1,389 | 1,084 | 1,000 | 1,389 | 1,506 | 1,017 | 0,962 | 1,000 | 1,017 | 0,979 |
| 91 | 0,904 | 0,692 | 1,000 | 0,904 | 0,625 | 0,558 | 0,955 | 1,000 | 0,558 | 0,533 | 0,711 | 0,813 | 1,000 | 0,711 | 0,577 |
| 92 | 1,532 | 1,214 | 1,335 | 1,147 | 1,859 | 0,977 | 0,980 | 1,000 | 0,977 | 0,958 | 1,223 | 1,091 | 1,156 | 1,059 | 1,334 |
| 93 | 1,000 | 1,207 | 1,000 | 1,000 | 1,207 | 1,000 | 0,719 | 1,000 | 1,000 | 0,719 | 1,000 | 0,931 | 1,000 | 1,000 | 0,931 |
| 94 | 1,058 | 1,187 | 1,000 | 1,058 | 1,256 | 1,000 | 0,887 | 1,000 | 1,000 | 0,887 | 1,028 | 1,026 | 1,000 | 1,028 | 1,055 |
| 95 | 1,764 | 0,927 | 1,478 | 1,194 | 1,636 | 1,116 | 0,964 | 1,000 | 1,116 | 1,076 | 1,403 | 0,946 | 1,216 | 1,154 | 1,327 |
| 96 | 1,000 | 0,922 | 1,000 | 1,000 | 0,922 | 0,755 | 0,970 | 0,898 | 0,841 | 0,733 | 0,869 | 0,946 | 0,948 | 0,917 | 0,822 |
| 97 | 1,000 | 0,970 | 1,000 | 1,000 | 0,970 | 1,000 | 0,975 | 1,000 | 1,000 | 0,975 | 1,000 | 0,972 | 1,000 | 1,000 | 0,972 |
| 98 | 1,136 | 1,040 | 0,964 | 1,178 | 1,180 | 1,165 | 1,066 | 1,175 | 0,991 | 1,242 | 1,150 | 1,053 | 1,064 | 1,081 | 1,211 |
| 99 | 0,633 | 0,952 | 0,707 | 0,895 | 0,602 | 1,265 | 1,019 | 1,415 | 0,894 | 1,288 | 0,895 | 0,985 | 1,000 | 0,895 | 0,881 |
| 100 | 1,000 | 0,991 | 1,000 | 1,000 | 0,991 | 1,000 | 1,212 | 1,000 | 1,000 | 1,212 | 1,000 | 1,096 | 1,000 | 1,000 | 1,096 |
| 101 | 1,000 | 1,113 | 1,000 | 1,000 | 1,113 | 1,000 | 1,067 | 1,000 | 1,000 | 1,067 | 1,000 | 1,090 | 1,000 | 1,000 | 1,090 |
| 102 | 0,486 | 0,941 | 1,012 | 0,480 | 0,458 | 0,891 | 1,193 | 1,000 | 0,891 | 1,063 | 0,658 | 1,060 | 1,006 | 0,654 | 0,698 |
| 103 | 0,816 | 0,825 | 1,000 | 0,816 | 0,674 | 1,044 | 1,030 | 1,000 | 1,044 | 1,076 | 0,923 | 0,922 | 1,000 | 0,923 | 0,851 |
| 104 | 1,032 | 0,985 | 1,045 | 0,987 | 1,016 | 1,029 | 1,052 | 1,000 | 1,029 | 1,082 | 1,030 | 1,018 | 1,022 | 1,008 | 1,049 |
| 105 | 0,850 | 1,018 | 1,000 | 0,850 | 0,865 | 2,190 | 0,870 | 1,000 | 2,190 | 1,906 | 1,364 | 0,941 | 1,000 | 1,364 | 1,284 |
| 106 | 1,143 | 1,281 | 1,168 | 0,978 | 1,465 | 1,517 | 0,846 | 1,739 | 0,873 | 1,283 | 1,317 | 1,041 | 1,425 | 0,924 | 1,371 |
| 107 | 0,782 | 1,169 | 0,770 | 1,016 | 0,914 | 1,409 | 0,996 | 1,240 | 1,136 | 1,403 | 1,050 | 1,079 | 0,977 | 1,074 | 1,133 |
| 108 | 0,878 | 0,730 | 1,000 | 0,878 | 0,641 | 0,786 | 1,041 | 1,000 | 0,786 | 0,818 | 0,830 | 0,872 | 1,000 | 0,830 | 0,724 |
| 109 | 0,903 | 0,953 | 1,000 | 0,903 | 0,861 | 0,885 | 1,101 | 1,000 | 0,885 | 0,974 | 0,894 | 1,024 | 1,000 | 0,894 | 0,916 |
| 110 | 1,000 | 0,580 | 1,000 | 1,000 | 0,580 | 1,000 | 0,638 | 1,000 | 1,000 | 0,638 | 1,000 | 0,609 | 1,000 | 1,000 | 0,609 |
| 111 | 1,248 | 1,132 | 1,000 | 1,248 | 1,413 | 1,000 | 1,026 | 1,000 | 1,000 | 1,026 | 1,117 | 1,078 | 1,000 | 1,117 | 1,204 |
| 112 | 1,000 | 1,300 | 1,000 | 1,000 | 1,300 | 1,000 | 0,825 | 1,000 | 1,000 | 0,825 | 1,000 | 1,036 | 1,000 | 1,000 | 1,036 |
| 113 | 1,000 | 1,624 | 1,000 | 1,000 | 1,624 | 1,000 | 0,551 | 1,000 | 1,000 | 0,551 | 1,000 | 0,946 | 1,000 | 1,000 | 0,946 |
| 114 | 0,974 | 0,821 | 0,999 | 0,975 | 0,800 | 1,026 | 1,044 | 1,001 | 1,026 | 1,072 | 1,000 | 0,926 | 1,000 | 1,000 | 0,926 |
| 115 | 0,668 | 0,725 | 0,872 | 0,766 | 0,485 | 0,909 | 1,012 | 0,863 | 1,053 | 0,919 | 0,779 | 0,857 | 0,867 | 0,898 | 0,667 |
| 116 | 0,999 | 1,158 | 1,000 | 0,999 | 1,157 | 0,914 | 1,170 | 1,000 | 0,914 | 1,069 | 0,955 | 1,164 | 1,000 | 0,955 | 1,112 |
| 117 | 1,136 | 1,002 | 1,023 | 1,110 | 1,138 | 0,921 | 0,944 | 1,000 | 0,921 | 0,869 | 1,023 | 0,972 | 1,012 | 1,011 | 0,995 |
| 118 | 1,181 | 1,176 | 1,000 | 1,181 | 1,388 | 1,175 | 0,880 | 1,000 | 1,175 | 1,034 | 1,178 | 1,017 | 1,000 | 1,178 | 1,198 |
| 119 | 1,141 | 1,089 | 1,127 | 1,013 | 1,243 | 1,000 | 0,937 | 1,000 | 1,000 | 0,937 | 1,068 | 1,010 | 1,061 | 1,006 | 1,079 |
| 120 | 0,336 | 0,632 | 0,503 | 0,668 | 0,213 | 1,182 | 0,997 | 1,173 | 1,007 | 1,178 | 0,630 | 0,794 | 0,769 | 0,820 | 0,501 |
| 121 | 1,111 | 1,018 | 1,111 | 1,000 | 1,131 | 1,000 | 1,292 | 1,000 | 1,000 | 1,292 | 1,054 | 1,147 | 1,054 | 1,000 | 1,209 |
| 122 | 1,000 | 1,073 | 1,000 | 1,000 | 1,073 | 1,000 | 0,922 | 1,000 | 1,000 | 0,922 | 1,000 | 0,995 | 1,000 | 1,000 | 0,995 |
| 123 | 1,342 | 0,994 | 1,315 | 1,021 | 1,334 | 0,788 | 1,097 | 0,830 | 0,949 | 0,864 | 1,028 | 1,044 | 1,045 | 0,984 | 1,074 |
| 124 | 1,561 | 1,043 | 1,264 | 1,235 | 1,627 | 0,913 | 0,908 | 1,000 | 0,913 | 0,830 | 1,194 | 0,973 | 1,124 | 1,062 | 1,162 |
| 125 | 0,922 | 1,012 | 0,965 | 0,956 | 0,933 | 1,464 | 1,027 | 1,360 | 1,077 | 1,504 | 1,162 | 1,019 | 1,145 | 1,014 | 1,185 |
| 126 | 0,908 | 1,028 | 0,952 | 0,954 | 0,934 | 0,868 | 0,953 | 0,804 | 1,080 | 0,828 | 0,888 | 0,990 | 0,875 | 1,015 | 0,879 |
| 127 | 1,000 | 1,254 | 1,000 | 1,000 | 1,254 | 0,941 | 0,553 | 1,000 | 0,941 | 0,521 | 0,970 | 0,833 | 1,000 | 0,970 | 0,808 |
| 128 | 1,238 | 0,837 | 1,000 | 1,238 | 1,036 | 0,865 | 1,029 | 1,000 | 0,865 | 0,890 | 1,035 | 0,928 | 1,000 | 1,035 | 0,961 |
| 129 | 1,339 | 0,934 | 1,311 | 1,022 | 1,250 | 1,151 | 0,948 | 1,190 | 0,968 | 1,091 | 1,242 | 0,941 | 1,249 | 0,994 | 1,168 |
| 130 | 1,000 | 0,989 | 1,000 | 1,000 | 0,989 | 1,000 | 1,018 | 1,000 | 1,000 | 1,018 | 1,000 | 1,003 | 1,000 | 1,000 | 1,003 |
| 131 | 1,000 | 1,210 | 1,000 | 1,000 | 1,210 | 1,000 | 0,953 | 1,000 | 1,000 | 0,953 | 1,000 | 1,074 | 1,000 | 1,000 | 1,074 |
| 132 | 1,000 | 1,258 | 1,000 | 1,000 | 1,258 | 1,000 | 0,579 | 1,000 | 1,000 | 0,579 | 1,000 | 0,853 | 1,000 | 1,000 | 0,853 |
| 133 | 1,000 | 1,097 | 1,000 | 1,000 | 1,097 | 1,000 | 0,904 | 1,000 | 1,000 | 0,904 | 1,000 | 0,996 | 1,000 | 1,000 | 0,996 |
| 134 | 1,000 | 0,991 | 1,000 | 1,000 | 0,991 | 1,000 | 0,984 | 1,000 | 1,000 | 0,984 | 1,000 | 0,987 | 1,000 | 1,000 | 0,987 |
| 135 | 0,506 | 1,442 | 0,699 | 0,723 | 0,730 | 1,212 | 0,738 | 1,142 | 1,061 | 0,894 | 0,783 | 1,032 | 0,894 | 0,876 | 0,808 |
| 136 | 1,000 | 1,247 | 1,000 | 1,000 | 1,247 | 1,000 | 0,856 | 1,000 | 1,000 | 0,856 | 1,000 | 1,033 | 1,000 | 1,000 | 1,033 |
| 137 | 0,685 | 0,897 | 1,000 | 0,685 | 0,614 | 1,143 | 0,971 | 1,000 | 1,143 | 1,111 | 0,885 | 0,933 | 1,000 | 0,885 | 0,826 |
| 138 | 1,091 | 0,932 | 1,000 | 1,091 | 1,018 | 0,958 | 1,065 | 1,000 | 0,958 | 1,020 | 1,023 | 0,996 | 1,000 | 1,023 | 1,019 |
| 139 | 1,000 | 0,981 | 1,000 | 1,000 | 0,981 | 1,000 | 1,051 | 1,000 | 1,000 | 1,051 | 1,000 | 1,015 | 1,000 | 1,000 | 1,015 |
| 140 | 0,991 | 1,084 | 0,836 | 1,186 | 1,075 | 2,766 | 0,901 | 2,261 | 1,223 | 2,492 | 1,656 | 0,988 | 1,375 | 1,204 | 1,637 |
| 141 | 1,000 | 0,945 | 1,000 | 1,000 | 0,945 | 1,000 | 0,862 | 1,000 | 1,000 | 0,862 | 1,000 | 0,902 | 1,000 | 1,000 | 0,902 |
| 142 | 1,353 | 1,461 | 1,000 | 1,353 | 1,977 | 1,000 | 0,930 | 1,000 | 1,000 | 0,930 | 1,163 | 1,166 | 1,000 | 1,163 | 1,356 |
| 143 | 0,600 | 1,044 | 0,732 | 0,820 | 0,626 | 1,082 | 0,967 | 1,005 | 1,076 | 1,045 | 0,806 | 1,004 | 0,858 | 0,939 | 0,809 |
| 144 | 1,000 | 1,215 | 1,000 | 1,000 | 1,215 | 1,000 | 1,722 | 1,000 | 1,000 | 1,722 | 1,000 | 1,446 | 1,000 | 1,000 | 1,446 |
| 145 | 1,000 | 1,054 | 1,000 | 1,000 | 1,054 | 0,919 | 1,144 | 1,000 | 0,919 | 1,051 | 0,958 | 1,098 | 1,000 | 0,958 | 1,052 |
| 146 | 1,000 | 0,856 | 1,000 | 1,000 | 0,856 | 1,000 | 1,014 | 1,000 | 1,000 | 1,014 | 1,000 | 0,932 | 1,000 | 1,000 | 0,932 |
| 147 | 1,043 | 1,138 | 1,000 | 1,043 | 1,187 | 1,000 | 1,033 | 1,000 | 1,000 | 1,033 | 1,021 | 1,084 | 1,000 | 1,021 | 1,107 |
| 148 | 0,948 | 1,051 | 1,000 | 0,948 | 0,996 | 1,046 | 1,069 | 1,000 | 1,046 | 1,118 | 0,996 | 1,060 | 1,000 | 0,996 | 1,055 |
| 149 | 1,000 | 1,005 | 1,000 | 1,000 | 1,005 | 0,359 | 0,996 | 0,520 | 0,692 | 0,358 | 0,600 | 1,001 | 0,721 | 0,832 | 0,600 |
| 150 | 1,000 | 0,896 | 1,000 | 1,000 | 0,896 | 1,000 | 0,997 | 1,000 | 1,000 | 0,997 | 1,000 | 0,945 | 1,000 | 1,000 | 0,945 |
| 151 | 1,000 | 0,894 | 1,000 | 1,000 | 0,894 | 1,000 | 0,944 | 1,000 | 1,000 | 0,944 | 1,000 | 0,919 | 1,000 | 1,000 | 0,919 |
| 152 | 1,000 | 1,075 | 1,000 | 1,000 | 1,075 | 1,000 | 0,987 | 1,000 | 1,000 | 0,987 | 1,000 | 1,030 | 1,000 | 1,000 | 1,030 |
| 153 | 1,000 | 1,675 | 1,000 | 1,000 | 1,675 | 1,000 | 0,478 | 1,000 | 1,000 | 0,478 | 1,000 | 0,895 | 1,000 | 1,000 | 0,895 |
| 154 | 1,269 | 1,180 | 1,064 | 1,193 | 1,498 | 1,000 | 0,971 | 1,000 | 1,000 | 0,971 | 1,127 | 1,070 | 1,031 | 1,092 | 1,206 |
| 155 | 1,000 | 0,955 | 1,000 | 1,000 | 0,955 | 1,000 | 1,215 | 1,000 | 1,000 | 1,215 | 1,000 | 1,077 | 1,000 | 1,000 | 1,077 |
| **mean** | **0,994** | **0,997** | **0,980** | **1,014** | **0,991** | **1,007** | **0,941** | **1,006** | **1,001** | **0,948** | 1,000 | 0,969 | 0,993 | 1,008 | 0,969 |
| Explanatory variables:  Effch – efficiency change  Techch – technology change  Pech – Pure technical efficiency change  Sech – scale efficiency change  Tfpch – Total factor productivity change  **Note that column “effch” is given by the multiplication of column “pech” and column “sech”, explaining the decomposition of technical efficiency change into change due to pure technical efficiency and scale efficiency.  **Note that column “tfpch” is given by the multiplication of column “effch” and column “techch”, explaining the decomposition of total productivity factor change into change due to technical efficiency and technology | | | | | | | | | | | | | | | |
